# Make Yourself at Home: Viral Hijacking of the PI3K/Akt Signaling Pathway

**DOI:** 10.3390/v5123192

**Published:** 2013-12-16

**Authors:** Nora Diehl, Heiner Schaal

**Affiliations:** Universitätsklinikum Düsseldorf, Institut für Virologie, Universitätsstraße 1, Düsseldorf 40225, Germany; E-Mail: nora.diehl@med.uni-duesseldorf.de

**Keywords:** PI3K/Akt signaling, viral entry, alternative splicing, mTOR, apoptosis

## Abstract

As viruses do not possess genes encoding for proteins required for translation, energy metabolism or membrane biosynthesis, they are classified as obligatory intracellular parasites that depend on a host cell to replicate. This genome limitation forces them to gain control over cellular processes to ensure their successful propagation. A diverse spectrum of virally encoded proteins tackling a broad spectrum of cellular pathways during most steps of the viral life cycle ranging from the host cell entry to viral protein translation has evolved. Since the host cell PI3K/Akt signaling pathway plays a critical regulatory role in many cellular processes including RNA processing, translation, autophagy and apoptosis, many viruses, in widely varying ways, target it. This review focuses on a number of remarkable examples of viral strategies, which exploit the PI3K/Akt signaling pathway for effective viral replication.

## 1. Introduction

The PI3K/Akt signaling pathway controls numerous cellular processes such as glucose metabolism, protein synthesis, and proliferation. Whereas the basal activity ensures cell survival, inactivation of PI3K/Akt signaling results in apoptosis. For an infected organism, apoptosis represents an effective antiviral instrument which is simple but highly effective. Thus, in order to secure its own replication, the virus must prevent or delay apoptosis. This may be achieved by maintaining the basal stimulatory activity of the PI3K/Akt signaling pathway. Since disabling host cell apoptosis is likely to be an obligatory step in the viral life cycle [1], regulation of the PI3K/Akt signaling pathway by viruses is quite likely to have had a considerable evolutionary impact. In response to this viral hijacking of key cellular processes, the PI3K/Akt pathway appears to be involved in the host cell immune response as a form of “adaptive strategy” to counteract viral invasion. Thus, when apoptosis is blocked by the virus, the PI3K/Akt signaling pathway induces expression of interferon-responsive genes [2,3,4,5,6]. Nevertheless, as a large number of viruses rely on PI3K activity for their replication, there would appear to be a greater benefit for the virus in activating rather than suppressing the PI3K/Akt signaling pathway.

### PI3K/Akt Signaling

The family of phosphoinositide 3-kinases (PI3Ks) is divided into three classes, based on (i) subcellular distribution; (ii) activating signals; and (iii) substrate specificity (for review see [7]). The best studied and presumably most relevant class when concerning viral targeting is the class I of PI3Ks, the members of which are composed of a regulatory (p85) and a catalytic subunit (p110) (for review see [8]).

**Figure 1:**
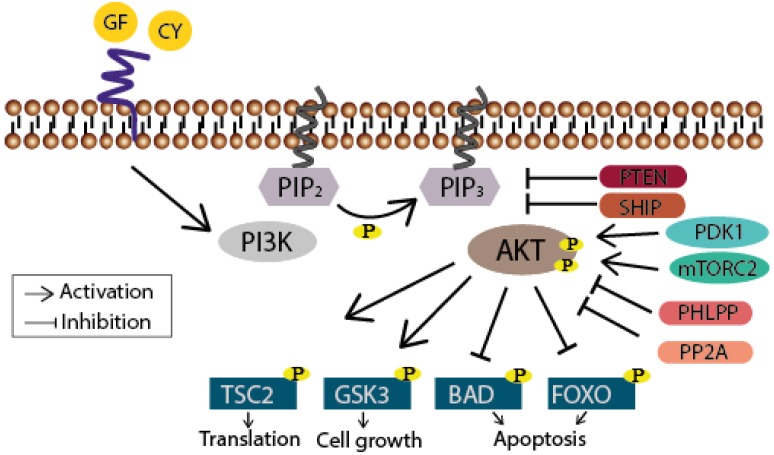
Schematic drawing of the PI3K/Akt signaling pathway. The phosphatidylinositol 3-kinase (PI3K) is activated through receptor-binding (receptor tyrosine kinases (RTKs) or G protein-coupled receptors (GPCR), sidled blue line) by growth factors (GF) or cytokines (CY) resulting in phosphorylation of PIP_2_. PIP_3_ subsequently serves as a second messenger allowing the binding of pleckstrin homology domain-containing proteins like Akt. Thereby the latter undergoes conformational changes leading to its phosphorylation and activation by PDK1 and mTORC2. Akt finally participates in the regulation of cellular processes like translation, cell growth and apoptosis by phosphorylating further proteins. Termination of the signaling cascade can either occur through the dephosphorylation of PIP_3_ by the phosphatase PTEN or SHIP, or further downstream through the dephosphorylation of Akt by PHLPP or PP2A.

When growth factors or cytokines are sensed by their receptor tyrosine kinases (RTKs) or G protein-coupled receptors (GPCRs), PI3K phosphorylates the 3-hydroxyl group of the inositol ring of membrane bound phosphatidylinositol (PtdIns) lipid substrates, generating phosphatidylinositol-3,4,5-trisphosphates (PIP_3_), which subsequently serve as docking stations for proteins that harbor lipid binding domains [8] (Figure 1). The most prominent effector of PI3K is the serine/threonine kinase Akt (for review see [9]). After binding of its pleckstrin homology (PH) domain to PIP_3_, Akt becomes phosphorylated at Thr308 by the phosphoinositide-dependent kinase 1 (PDK1) and at Ser473 by mammalian target of rapamycin complex 2 (mTORC2) leading to its full activation. Notably, stimulus-dependent, as in the case of the DNA-damage response, Ser473 is phosphorylated by the DNA-dependent protein kinase (DNA-PK) [10,11]. Finally, several downstream targets like tuberous sclerosis protein 2 (TSC2; translation), glycogen synthase kinase 3 (GSK3; cell growth), or Bcl-2-associated death promoter (BAD; cell survival) and forkhead box protein (FOXO; cell survival) are altered in their function through the Akt-mediated phosphorylation. Termination of this signaling cascade can occur through the dephosphorylation of PIP_3_ either by the phosphatase and tensin homolog (PTEN) or the Src homology domain 2 containing inositol-5-phosphatase (SHIP). Akt activity can also be directly abolished through its dephosphorylation carried out by phosphatases like the protein phosphatase 2A (PP2A) or the H domain and leucine-rich repeat protein phosphatase (PHLPP) [12,13].

## 2. Viral Entry

In the course of the virus life cycle, viral attachment to the host cell is the earliest time point at which host signaling pathways are activated. Interaction with the viral receptor embedded within the host cell membrane activates and triggers a signaling cascade coincident with viral entry to set the stage for a favorable cellular environment for viral needs. For example, within a minute after HIV-1 exposure, more than 200 phosphorylation sites are modified in T-cells, with the potential to alter several cellular processes immediately after infection and thus to support viral replication [14].

**Figure 2:**
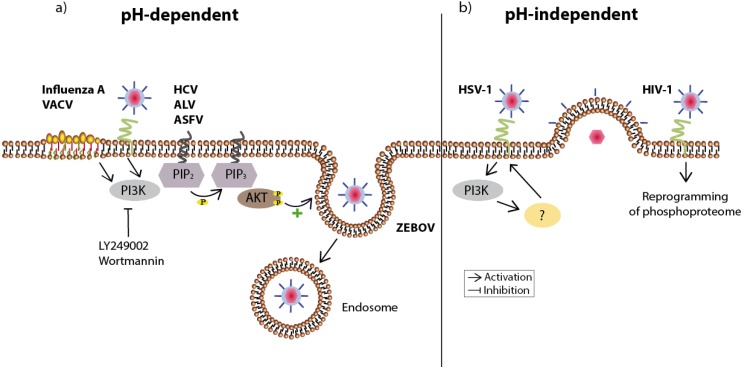
Overview of viruses utilizing the PI3K/Akt signaling pathway during host cell entry. (**a**) Viruses using the pH-dependent route to enter the host cell are engulfed in endosomes and released inside the cell. Attachment of influenza A or vaccinia virus (VACV) results in a clustering of lipid rafts at the cell surface (here: accentuated part of the membrane), which is followed by PI3K/Akt-supported endocytosis. Inhibition of PI3K (LY294002 or Wortmannin) reduces the infection by both viruses. The endosomal entry of Hepatitis C virus (HCV), Avian leucosis virus (ALV), and African swine fever virus (ASFV) is also diminished after inhibition of this signaling pathway. In the case of Zaire Ebola virus (ZEBOV) the signaling pathway is important for further trafficking into the cell as inhibition traps virus particles in vesicular compartments; (**b**) Likewise, the pH-independent entry of herpes simplex virus 1 (HSV-1)—carried out through fusion of the viral envelope with the plasma membrane—relies on PI3K activity. The receptor binding of human immunodeficiency virus (HIV-1) already affects several hundred intracellular phosphorylation events, which potentially support the viral life cycle. The respective viral receptors are shown in green.

In general, enveloped viruses can enter their host cell via two routes (for review see [15]). Using a pH-independent route, the virus attaches to its host cell and by interacting with its corresponding host-cell receptor induces conformational changes of the viral glycoprotein causing the fusion of the viral envelope with the plasma membrane of the target cell. This directly leads to the delivery of the capsid into the cytoplasm followed by its uncoating. In a second route which is pH-dependent, the virus enters the cell via endocytosis, a process essential in eukaryotes to internalize extracellular molecules. In this process the virus is first engulfed into early endosomes, which later on mature into late endosomes with a low pH, inducing the necessary conformational change of the viral glycoprotein to induce fusion of the viral and endosomal membranes. Non-enveloped viruses can also enter through endocytotic mechanisms, however they cross this internal membrane by pore formation or penetration of the membrane [15] (Figure 2).

In immunological terms, endocytosis represents an inconspicuous way of entering a host cell by delaying the immune recognition of infection but still guarantees a subsequent ferry to subcellular sites of viral replication (for review see [16]). The success of this pathway may explain its use by a multitude of viruses. Importantly, the attachment of the virus to the host cell membrane not only seems to be a suitable instance for activating the PI3K/Akt signaling pathway for already triggering a desired cellular surrounding; rather it seems that the pathway is directly involved in the entry of various viruses by facilitating endocytotic uptake.

This is the case for the enveloped influenza A virus, whose binding to its sialic acid receptor results in the clustering of plasma membrane lipid rafts [17] (Figure 2). These are small domains within the plasma membrane enriched for cholesterols and glycosphingolipids [18]. As they contain different sets of proteins—mainly signaling molecules—lipid rafts are thought to be involved in signal transduction (for review see [19]). The clustering of such lipid rafts after influenza A attachment subsequently leads to the activation of at least two RTKs followed by signaling events through the PI3K/Akt signaling pathway, which promote viral internalization [20], supported by the cross-interaction of PI3K with the Ras signaling pathway [21]. Moreover, acidification of the endosomal interior, necessary for fusion of the viral and endosomal membranes is in part controlled by ERK and PI3K by upregulating the vacuolar H^+^-ATPase activity. Based on immunoprecipitation experiments using extracts from the epithelial cell lines MDCK and A549, direct interactions between the phosphorylated forms of ERK 1/2 and the PI3K p85α subunit with the E subunit of this proton pump have been shown [22].

Infection with the enveloped vaccinia virus (VACV) also causes a clustering of lipid rafts at the host plasma membrane, leading to co-localization of virus and raft-associated integrin β, which finally activates the PI3K/Akt signaling pathway to support endocytosis of the virus [23,24].

The enveloped Zaire Ebola virus (ZEBOV) likewise enters the host via the cellular endocytotic pathway. In this case, the PI3K/Akt signaling pathway appears to be essential for a step immediately after viral uptake, as inhibition of the cascade leaves virus particle stuck in vesicular compartments, compatible with an arrest of further trafficking within the cell [25]. Recently an early and transient activation of Akt by the enveloped hepatitis C virus (HCV) was also reported, which is most certainly achieved through an interaction between the viral envelope protein E2 and its cellular co-receptors CD81 and claudin-1. This early activation is essential for HCV entry, whereas later in infection Akt activity seems dispensable [26].

Infection with the enveloped, acute-transforming oncogenic retrovirus, avian leucosis virus (ALV), results in a transient, PI3K-dependent phosphorylation of Akt as early as 15 minutes post infection. Moreover, treatment of cells with either PI3K inhibitor LY294002 or Wortmannin prior to infection significantly reduces viral replication in a dose-dependent manner [27], pointing to a role of the pathway during the entry mechanism.

The enveloped herpes simplex virus type-1 (HSV-1) attaches to the cell by heparan sulfates leading to the fusion of the viral envelope with the plasma membrane. In addition to this, the infection is accompanied by cytoskeletal changes facilitating filopodia formation and thus membrane fusion. The filopodia formation leads to the activation of Rho-GTPase signaling [28]. The PI3K signaling pathway seems to be involved in both, as inhibition prior to infection blocks Rho-GTPase signaling, reduces filopodia formation and thus RhoA activity [29]. Activation of signaling events typical of macropinocytosis also seems to be activated directly, following cellular entry of the highly pathogenic African swine fever virus. This virus not only induces two critical effectors, Pak1 and Rac1, which link Rho-GTPases to cytoskeleton reorganization and thus membrane perturbations and actin remodeling, but also to the EGFR and PI3K/Akt pathway. Consequently, specific inhibitors of EGFR, *i.e.*, 324674 or the phytoestrogen genistein, as well as inhibition of PI3K with LY294002, analyzed by the phosphorylation status of its specific substrate PIP_2_, efficiently reduced viral uptake [30].

Although the molecular mechanisms within the PI3K pathway leading to endocytotic uptake still remain elusive in many cases, the usage of this pathway for assisting viral uptake seems to be a widespread viral strategy [20,21,23,25,26,27,29,30] (Figure 2).

## 3. Pre-mRNA Splicing

Eukaryotic pre-mRNA splicing is a process during which introns are removed and the flanking exons ligated to form the mature mRNA. Alternative splicing enables differential usage of splice sites, thus generating various transcript isoforms originating from one genetic template and increasing the proteomic diversity (for recent review see [31]).

The usage of alternative splice sites is regulated by *trans*-acting splicing factors like SR (serine arginine rich) proteins or hnRNPs (heterogeneous nuclear ribonucleoproteins) which bind to *cis*-regulatory elements in the neighborhood of the splice sites (for review see [32]). Both classes of these splicing regulators show dual and antagonistic functionality in a strict position-dependent manner [33]. Accordingly, expression, positioning and activity of these proteins are critical to splice site selection. A growing body of evidence reveals the contribution of signaling pathways controlling alternative splicing by transmitting incoming signals to the splicing machinery. This allows a faster reaction to changing environmental conditions as transcript variants of different stability or protein isoforms can be generated in the absence of a new round of transcription.

**Figure 3:**
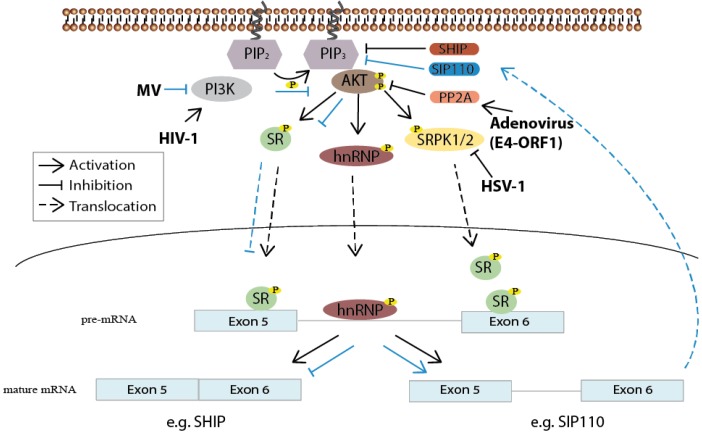
PI3K/Akt contribution to the regulation of alternative splicing and viruses utilizing the pathway for splicing regulation. After PI3K-mediated phosphorylation, Akt indirectly—through SRPK1/2—or directly phosphorylates SR proteins resulting in their translocation into the nucleus where they bind to corresponding pre-mRNAs. hnRNP proteins can also be targeted by Akt-mediated phosphorylation. Measles virus (MV) interferes with PI3K activity, leading to altered SR protein phosphorylation and subsequently to the alternative splicing of a constitutively active isoform of the PIP_3_ phosphatase SHIP, namely SIP110, which results in the down regulation of Akt activity (this route is marked with blue arrows). The adenovirus E4-ORF1 protein and herpes simplex virus 1 (HSV-1) control SR protein activity in support of viral replication either through modulating PP2A or SRPK1 activity. Human immunodeficiency virus (HIV-1) seems to depend on PI3K activity for accurate splicing of its own mRNA.

In response to extracellular stimuli, Akt indirectly acts on splicing regulation by phosphorylating serine/threonine-protein kinase 2 (SRPK2) or by inducing the autophosphorylation of serine/threonine-protein kinase 1 (SRPK1), leading to the translocation of both kinases into the nucleus thus modifying SR protein activities [34,35]. Furthermore, SRSF1, SRSF7 and SRSF5 can directly be phosphorylated by Akt itself [36,37,38] (Figure 3).

However not only SR proteins but also hnRNP L was demonstrated recently to be phosphorylated in an Akt-dependent manner leading to the exclusion of 4 exons from the caspase-9 pre-mRNA. Skipping of exons 3, 4, 5, and 6 results in the anti-apoptotic caspase-9b isoform, whose protein product interferes with the formation of a functional Apaf-1-caspase-9 complex [39,40]. Inclusion of these four exons, on the other hand, led to the synthesis of the antagonistic protein isoform of caspase-9, which is necessary for induction of apoptosis by the pro-apoptotic Bcl-2 family members [39], calling for a precise regulation of these particular splice sites. The study elegantly refutes the dogma that the PI3K/Akt pathway only modulates alternative splicing through the regulation of SR proteins. Concerning apoptosis, Acinus—a component of the apoptosis-and splicing-associated protein complex (ASAP) which is present in functional spliceosomes [41,42]—was also shown to be directly phosphorylated by Akt, at least during chromatin condensation, leading to the inhibition of its pro-apoptotic function [43]. This might be a clue to the general role of Akt in regulating the function of the splicing relevant ASAP complex, once again emphasizing the close relationship between splicing and apoptosis. These examples underline the significance of intermediated kinases, like PI3K and Akt, in transforming incoming signals via the regulation of alternative splicing.

Viruses also profit from the presence of multiple cellular protein isoforms and apparently are able to regulate their synthesis. In T-cells, for example, the single-stranded RNA measles virus blocks PI3K/Akt activity, and thereby down regulates SR protein phosphorylation and their translocation into the nucleus. As a result, a constitutively active splicing isoform of the lipid phosphatase SHIP, termed SIP110, containing intronic sequences between exon 5 and 6, is expressed. This, in turn, leads to an increase in PIP_2_ levels and consequently to a down regulation of activated PI3K levels, necessary for activating T-cell proliferation [44]. In this particular case, the virus inhibits the signaling pathway instead of stimulating it, emphasizing the ability of viruses to trigger cascades in either direction.

Recently, a global role of the PI3K signaling pathway in T-cell activation was revealed [45]. Using exon arrays, Riedel *et al*. [45] showed that the inhibition of PI3K interferes with T-cell activation through altered mRNA expression levels and alternative splicing patterns.

Expanding their coding potential, many viruses take advantage of the host splicing machinery to ensure the production of their own protein diversity and to regulate the different stages of infection by temporally expressing transcripts. Adenoviruses and HSV-1, both containing a double-stranded DNA genome, seem to manipulate mRNA splicing in favor of viral gene expression and replication. Both viruses do so via hypophosphorylation of particularly two SR proteins, SRSF1, SRSF9 (Adenovirus) and SRSF3, SRSF5 (HSV-1), by modifying either PP2A or SRPK1activity [46,47] (Figure 3).

In monocyte-derived macrophages, the expression levels of hnRNP A1, hnRNP A2/B1 and hnRNP H decrease upon human immunodeficiency virus 1 (HIV-1) infection, reaching levels comparable to untreated control cells 2 weeks after infection. SRSF2 levels, on the other hand, are increased 2–3 weeks post infection and then decline again [48]. In CD4^+^ T-cells, the phosphorylation status of six SR proteins—including SRSF2—and five other splicing related proteins immediately changed after HIV-receptor interaction. Furthermore, most of these proteins were crucial for balanced HIV-1 transcripts and thus p24 levels [14].

Interestingly, the PI3K signaling pathway seems to be involved in HIV-1-mediated phosphorylation of SR proteins, as inhibition of the kinase results in an altered phosphorylation pattern of some SR proteins and concomitantly in an alternative splicing pattern of HIV-1 mRNAs, accompanied by a significant reduction of viral replication [49]. These observations suggest an HIV-1-induced PI3K/Akt-dependent modulation of splicing regulation, which seems to be essential for the viral life cycle. In sum, these results emphasize the importance of splicing regulatory proteins for proper HIV-1 splicing, yet the impact on cellular alternative splicing and reprogramming in favor of viral replication has not yet been examined. It will take further effort to uncover the number of viruses targeting the cellular splicing machinery to support and modulate gene expression. The identification of the cellular players concerned will likewise help decipher the biological relevance of the PI3K/Akt pathway regarding the regulation of pre-mRNA processing.

## 4. Cell Survival

**Figure 4:**
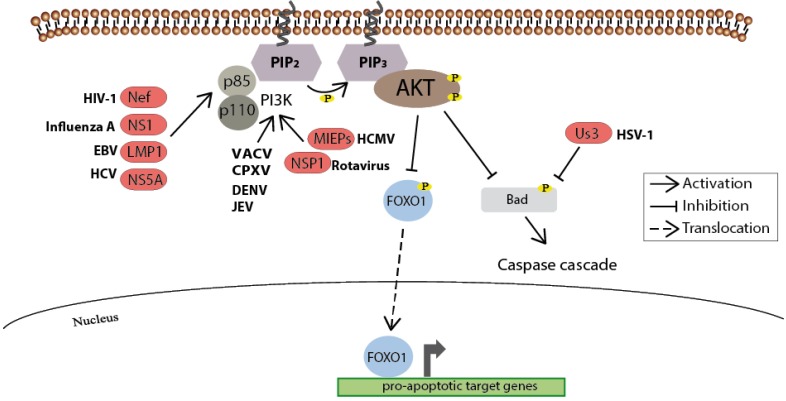
As a key regulator of cellular survival the PI3K/Akt signaling pathway is often triggered by viruses. Upon incoming survival signals, the Akt-mediated inhibitory phosphorylation of pro-apoptotic molecules such as BAD prevents the induction of the caspase cascade. In addition, the inhibitory phosphorylation of the transcription factor FOXO1 by Akt blocks its translocation into the nucleus and thus prevents the expression of pro-apoptotic genes. A variety of viral proteins steps into this regulatory network to assure cell survival, and thus maintaining the cellular environment for viral replication. Known viral proteins interacting with the cellular member of the signaling pathways are highlighted in red.

Maintaining cell survival by acting downstream of growth factors is another key function of the Akt kinase (for review see [50] and [51]). For this purpose Akt interferes with pro-apoptotic molecules to inhibit their activity either through a direct phosphorylation of the regulators as in the case of several Bcl-2 homology domain 3 (BH3)-only proteins (e.g., BAD), or indirectly through the phosphorylation of certain transcription factors such as FOXO1 which thereupon translocate out of the nucleus and thus can no longer promote the transcription of their pro-apoptotic target genes [52,53] (Figure 4).

As survival of the host cell ensures maintenance or replication of the intracellular viruses until the infectious particles are assembled and spread throughout the organism, it is of no surprise that quite a lot of work has been done to investigate virus-induced Akt-dependent anti-apoptotic mechanisms.

An HIV-1 Nef-mediated inhibition of PI3K activation through preventing the interaction of PI3K with the platelet-derived growth factor receptor was demonstrated by Graziani and colleagues in 1996 [54]. Later a Nef-mediated activation of PI3K, which resulted in the inhibitory phosphorylation of the pro-apoptotic factor BAD blocking premature apoptosis in T-cells, was observed. Although the decline of T-cells is characteristic of progressive HIV infection, this early delay may ensure cellular survival until viral particles are assembled and released from the cell [55]. The authors suggest that these conflicting observations could be the result of a different cellular localization of Nef during the experiments. Graziani and colleagues used a stable cell line expressing Nef mostly in the cytoplasm and not directly at the plasma membrane [54], at which Wolf and coworkers showed the protein to be necessary for the activation of PI3K signaling [55]. Although ectopic expression of viral genes might be a useful, and sometimes indispensable tool to unravel protein interactions, there is also an inherent risk of missing out on the temporal and spatial aspects of kinase activation during viral replication.

Several groups have established that the influenza A non-structural protein 1 (NS1), known for its role in suppressing host immune responses [56], directly activates the PI3K/Akt pathway to secure anti-apoptotic signaling by interacting with the PI3K regulatory subunit p85 [57,58,59,60]. In contrast, Jackson and coworkers suggested an NS1-mediated, but PI3K-independent, prevention of apoptosis as NS1 mutant viruses induced apoptosis, while FCS-activated Akt was not able to overcome this effect [61]. Even though strain-specific viral requirements for NS1-activated PI3K have been discovered, mere strain-specificity cannot explain the observed differences concerning the role of PI3K in NS1-mediated anti-apoptotic signaling, since both labs used the same influenza A strain [62,63]. However, Ayllon and colleagues, using mouse-adapted viruses, demonstrated *in vivo* subtle differences in NS1 protein localizations depending on the NS1 isoform, which only differs in seven amino acids between the two strains, A/Puerto Rico/8/34 (PR8) and A/WSN/33. Applying a cell-based assay, they observed that PR8/NS1-induced PIP_3_, the target of PI3K, appeared to accumulate in microdomains whereas WSN/NS1-induced PIP_3_ was broadly distributed throughout the plasma membrane, which may explain that different NS1 variants define intracellular sites of PI3K activation [62,63]. To entirely decode how influenza A virus impacts the PI3K signaling pathway, the authors suggest carefully selecting the viral strain, host-cell type, time post-infection and the PI3K isotype to be used in further experiments [62]. 

HSV-1, with a genome of approximately 150 kb encoding more than 70 proteins, is able to afford its own serine/threonine kinase. Nonetheless, early during HSV-1 infection, apoptosis is still blocked in an Akt-dependent manner until the virus has accumulated enough Us3 protein kinase to mimic Akt activity. However, in the absence of Us3, the virus appears to have evolved a backup mechanism to retain the activity of Akt thereby ensuring anti-apoptotic signaling [64]. Another herpes virus thought to manipulate the PI3K/Akt-signaling pathway is the human cytomegalovirus (HCMV). Both the HCMV major immediate-early proteins (MIEPs) and a constitutive active form of Akt can inhibit temperature-induced apoptosis in ts13 cells. Since this ability of the MIEPs to inhibit apoptosis is lost when PI3K/Akt signaling is inhibited by LY294002 and since MIEPs can activate Akt, it was concluded that the MIEPs induced anti-apoptotic activity is Akt-mediated [65].

A third member of the herpes virus family, Epstein-Barr virus (EBV), induces the PI3K/Akt pathway through the viral latent membrane protein 1 (LMP1), resulting in host-cell survival, which most probably contributes to EBV persistence in B cells necessitating sustained apoptotic inhibition [66]. Both viral EBV transcriptional activators, BZLF1 and BRLF1, can reactivate the lytic form of viral replication with BRLF1 acting in a PI3K/Akt-dependent manner as inhibition of PI3K abolishes BRLF1-induced transcriptional activation [67]. Recently, however, for two other herpes viruses, murine gamma herpesvirus-68 (MHV-68) and human herpesvirus-8/Kaposi’s sarcoma-associated herpesvirus (HHV8/KSHV), Peng and coworkers demonstrated that Akt promotes viral persistence by suppressing transcriptional reactivation of these viruses rather than reactivating lytic replication. In this case reactivation or the transition from latency to lytic replication is controlled by the viral transcription activator (RTA), an immediately-early (IE) gene, whose activity is negatively regulated by the PI3K/Akt pathway [68]. They observed enhanced MHV-68 production in permissive fibroblast after either LY294002 treatment in a dose-dependent manner or RNAi-mediated Akt1 silencing. For the various other capabilities of the virus to modulate host cell pathways for longer periods the reader is referred to the detailed review by Cooray [69].

Two members of the poxvirus family, VACV—which already uses the pathway to assist its endocytotic uptake [23]—and cowpox virus (CPXV), also hijack the PI3K/Akt signaling pathway to prevent apoptosis. Inhibition of Akt activity either by the pharmaceutical PI3K inhibitor LY294002 or by expressing a dominant-negative form of Akt reduces viral titers by up to 90%, corresponding to cleavage of caspase-3 and PARP as significant indicators of apoptotic cells [70].

In rotavirus infected cells an increased Akt phosphorylation depends on a direct interaction between the viral nonstructural protein NSP1 and PI3K. This interaction leads to the Akt-dependent inactivation of pro-apoptotic proteins on the one hand and the activation of NFkβ-dependent induction of anti-apoptotic genes on the other [71,72]. An association of these two survival pathways has been known for a long time [73] and Bagchi and coworkers suggest these to be partially overlapping or even cooperative in case of rotavirus infection [71]. Dengue virus and japanese encephalitis virus, both members of the single-stranded RNA flavivirus family, also make use of a PI3K-dependent blocking of apoptosis as early as 15 min after infection, indicating that the mere viral attachment leads to an activation of the signaling pathway [74]. However, treatment of infected cells with the PI3K inhibitors LY294002 or Wortmannin did not alter effective viral replication. Nonetheless the PI3K/Akt signaling appears to protect the infected cells from early apoptosis and thereby offers more time to generate virus progeny.

Besides its beneficial role during HCV entry [26], the PI3K/Akt pathway also mediates survival of the HCV-infected cells [75,76]. The replication of HCV is accompanied by the formation of lipid rafts, followed by an increased production of the proto-oncogene N-Ras, a known activator of the PI3K-signaling pathway [77]. This consequently results in Akt activity leading to protection against apoptosis. Interestingly, upon inhibition of either signaling molecules, the levels of HCV replication is raised. As this virus establishes chronic infection in most of the cases, Mannova and Beretta claimed that the activation of the Ras-PI3K-Akt signaling pathway may ensure both cellular survival and the maintenance of persistent infection by maintaining the virus production at a low level [76].

As respiratory syncytial virus (RSV), coxsackievirus B3 (CVB3), avian reovirus (ARV), rubella virus and SARS coronavirus also utilize the PI3K-mediated cell survival [78,79,80,81,82], this highlights the obvious advantage viruses enjoy from the prolonged cell survival.

## 5. Viral Translation

Protein synthesis requires many different enzymes, none of which are encoded by viral genomes. As viruses use the cellular translation machinery for protein synthesis, their mRNAs must efficiently be recognized by the cellular ribosomal 40S subunit, either in a cap-dependent or cap-independent manner.

**Figure 5:**
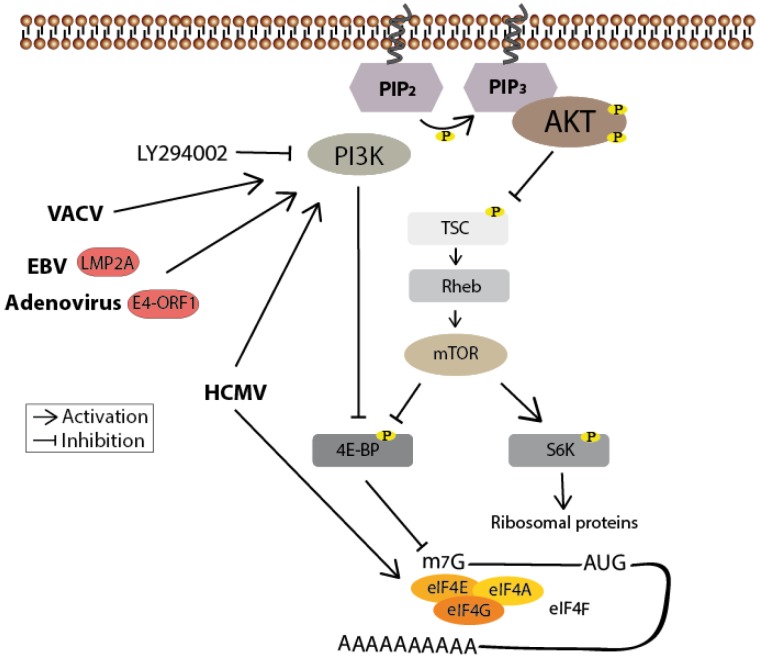
PI3K/Akt-mediated mTOR activation positively regulates protein synthesis. Inhibitory phosphorylation by Akt prevents TSC-mediated stimulation of the GTPase Rheb, leading to the activation of mTOR. Activated mTOR then phosphorylates 4E-BP, thereby enabling eIF4F cap-binding complex formation and thus cap-dependent translation. It also phosphorylates S6K to facilitate ribosome biogenesis. Human cytomegalovirus (HCMV)-mediated phosphorylation of the eIF4F subunit eIF4E is both mTOR- and PI3K-independent, whereas its phosphorylation of 4E-BP is dependent on PI3K. This is also the case for the vaccinia virus (VACV)-mediated 4E-BP phosphorylation. Epstein-Barr virus (EBV) and adenovirus on the other hand seem to regulate translation in a PI3K and mTOR-dependent manner. Known viral proteins interacting with the cellular member of the signaling pathways are highlighted in red.

One indirect downstream target of Akt is the kinase mTOR (mammalian target of rapamycin) within the mTOR complex 1 (for review see [83]). This kinase is necessary for the regulation of cap-dependent translation carried out by the initiation complex eIF4F. After its inhibitory phosphorylation by activated Akt kinase [84], the TSC is unable to stimulate the intrinsic GTPase activity of Rheb, which finally leads to the maintenance of high levels of GTP. GTP then removes FKBP38 from mTORC1, thus restoring the latters’ activity. Active mTOR in turn phosphorylates both the p70S6 kinase (S6K), which promotes translation elongation and ribosome biogenesis, and the eIF4E binding protein (4E-BP). Phosphorylated 4E-BP cannot prevent formation of the eIF4F complex—consisting of the cap-binding subunit eIF4E, the RNA helicase eIF4A and the scaffolding protein eIF4G—thus translation can be initiated by the recruitment of the preinitiation-complex to the 7-methylguanosine (m7G) cap of the 5' end of the mRNA [85] (Figure 5). Hence viruses, depending on cellular 5’cap-dependent translation, must establish a strategy to either maintain mTOR activity or directly ensure eIF4F complex formation.

HCMV-induced Akt phosphorylation seems to regulate, in addition to the prolonged cell survival [65], viral translation. Treatment of infected cells with the pharmaceutical PI3K inhibitor LY294002 reduces viral titers by more than 4 logs. Surprisingly, rapamycin (an mTOR inhibitor), as opposed to LY294002 treatment, does not diminish viral protein synthesis, indicating an HCMV-induced, mTOR-independent mechanism to maintain translation [86]. Later it was indeed found that HCMV phosphorylates two downstream targets of mTOR: the transcriptional repressor 4E-BP and the scaffolding protein eIF4G to maintain the activity of the eIF4F complex. Thereby, both phosphorylations are in fact mTOR-independent, whereas the phosphorylation of 4E-BP is PI3K-dependent, explaining the diminished viral protein synthesis after PI3K inhibition [87].

Additionally, HCMV enhances the activity of the cap-binding unit eIF4E [88] and also activates rictor, the kinase of Akt S473 [89]. Remarkably, both the raptor complex (mTORC1) and the rictor complex (mTORC2) can mediate an inhibitory phosphorylation of 4E-BP and a stimulatory phosphorylation of p70S6K. This indicates that upon HCMV infection the substrate specificity of rictor and raptor is modified, correlating with a beneficial rictor-mediated Akt activation.

Yet another member of the herpes virus family, EBV, affects, in addition to the regulation of apoptosis [66], the cellular translational machinery. The viral protein LMP2A activates mTOR1, most certainly via the PI3K/Akt signaling pathway since the activation is sensitive to the PI3K inhibitor Wortmannin. This leads to an enhanced disassociation of 4E-BP from eIF4E and thus to an activation of the cap-dependent translation [90].

VACV gains, besides the facilitated viral uptake and the prevention of apoptosis [23,70], a third benefit out of the PI3K/Akt signaling activation. The increased phosphorylation level of Akt post infection corresponds with a decreased level of repressor 4E-BP bound to eIF4E. Inhibition of the PI3K, but not the mTOR1 pathway, suppresses the production of VACV proteins, indicating an mTOR-independent but PI3K-dependent mechanism to regulate 4E-BP repressor activity, as in the case of HCMV [91]. Two of the human papillomavirus (HPV) early proteins, E6 and E7, not only activate Akt/mTOR signaling but also maintain the phosphorylation status by inhibiting the Akt phosphatase PP2A. This results in an inactivation of 4E-BP and in an activation of S6K, supporting viral cap-dependent protein synthesis [92,93]. These signaling events were recently also shown to be triggered by the HPV-mediated EGFR stimulation during viral entry [94].

Besides the modification of SR protein phosphorylation [46], adenoviruses also activate the mTOR1 pathway [95]. Two viral proteins, E4-ORF1 and E4-ORF4, function to mimic incoming signals to activate this pathway. Interestingly, the proteins seem to act via different mechanisms as only E4-ORF1 is necessary and sufficient for the PI3K-dependent activation of S6K, whereas E4-ORF4 acts independently of PI3K. Both proteins seem to collaborate to induce mTOR activity, independent of the stimulation by nutrients or growth factors.

Taken together, the manipulation of cap-dependent protein synthesis—irrespective of the involvement of mTOR1—is a common mechanism of viruses to guarantee an efficient translation of viral proteins.

## 6. Conclusions

**Figure 6:**
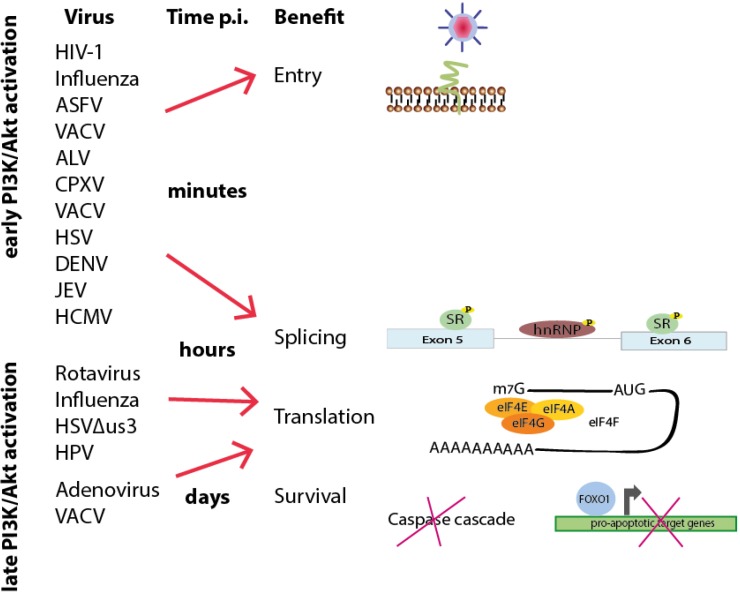
Time schedule of viral-induced interference with PI3K/Akt signaling based on the time point of activation and benefit during viral life cycles. Early activation of the PI3K/Akt signaling pathway during the first contact with the cell or a few minutes after entry can lead to both short-term cellular answers to facilitate viral entry and long-term reactions (lasting for hours or even days) to support viral and cellular splicing, viral translation and cell survival. Later activation, hours or days post-infection, can likewise promote such long-term effects.

In the past decades our knowledge of virus-induced modifications of host signaling pathways has grown rapidly and the diversity of viruses interfering with the PI3K/Akt pathway notably highlights the importance of this research field. Table 1 summarizes interacting viral proteins and Figure 6 additionally points out the remarkable temporal range of processes during viral life cycles affected by either transient or long-term activation of the PI3K pathway.

**Table 1:** Viruses and viral proteins (if known) that interact with the PI3K/Akt signaling pathway during different steps of the viral life cycle.

Viral Life Cycle	Virus	Protein	Function	Reference
**Entry**	Influenza	-	Virus internalization;Endosomal acidification	[20,22]
	VACV	-	Integrin β1-dependent virus entry	[23]
	HCV	E2	Facilitation of virus entry	[26]
	ALV	-	Facilitation of virus entry	[27]
	HSV-1	-	Filopodia formation; Fusion	[29]
	ASFV	-	Macropinocytosis	[30]
**pre-mRNA Splicing**	HIV-1		Alternative splicing of viral mRNAs	[49]
	Adenovirus	E4-ORF4	Dephosphorylation of SF2/ASF and SRp30c; splicing of viral mRNAs	[46]
	MV		T-cell proliferation	[44]
**Cell Survival**	HIV-1	Nef	Anti-apoptotic effects	[54,55]
	Influenza	NS1	Anti-apoptotic effects	[57,58,59]
	VACV	-	Anti-apoptotic effects	[70]
	HCV	NS5A	Anti-apoptotic effects; viral persistence	[75,76]
	CPXV	-	Anti-apoptotic effects	[70]
	DENV	-	Anti-apoptotic effects	[74]
	JEV	-	Anti-apoptotic effects	[74]
	HCMV	MIEPs	Anti-apoptotic effects	[65]
	Rotavirus	NSP1	Anti-apoptotic effects	[71]
	EBV	LMP1	Persistence	[66]
	RSV	-	Anti-apoptotic effects	[78]
	CVB3	-	Anti-apoptotic effects	[79]
	ARV	-	Anti-apoptotic effects	[80]
**Translation**	VACV	-	Translation initiation via activity of 4E-BP	[91]
	HCMV	-	Translation initiation via activity of 4E-BP	[87]
	Adenovirus	E4-ORF1	mTOR activation	[95]
	EBV	LMP2A	mTOR activation	[90]
	HPV	E6, E7	mTOR activation	[93]

In general, unraveling of signaling pathways is a challenging task, especially where viral interference occurs. The sensitive regulatory system can be easily disturbed by experimental conditions used to mimic the infection of a complex organism. In particular the cell lines used may omit the complex network of cross-talk between signaling pathways in a real infection situation. Moreover, many cancer T-cell lines, which are commonly utilized when concerning infections, often carry mutations in genes encoding members of the PI3K/Akt signaling pathway [96,97]. This is of no surprise bearing in mind the role of the pathway in the regulation of apoptosis and proliferation. Nevertheless, the growing body of evidence of the continuous interaction between the viral survival strategies and the host cell-induced defense mechanisms, such as apoptosis, not only helps us understand the viral strategy of taking over control of cellular processes, but also offers additional insights into general mechanisms of such processes. New methods like next generation sequencing and high throughput protein identification are promising tools to track the viral-host interactions and to monitor the impact on several levels of the host cell metabolism, finally supporting the development of antiviral therapies.
